# The aryl hydrocarbon receptor in liver injury: a double-edged sword in roles, mechanisms, and future perspectives

**DOI:** 10.3389/fphar.2025.1720827

**Published:** 2026-01-08

**Authors:** Yupeng Wang, Tianqi Ren, Yikun Zhang, Shuangxing Li, Yanwei Yang, Chao Wang, Xingchao Geng

**Affiliations:** 1 NHC Key Laboratory of Research on Quality and Standardization of Biotech Products and NMPA Key Laboratory for Quality Research and Evaluation of Biological Products, Institute for Biological Product Control, National Institutes for Food and Drug Control, Beijing, China; 2 School of Basic Medicine and Clinical Pharmacy, China Pharmaceutical University, Nanjing, China; 3 National Center for Safety Evaluation of Drugs, National Institutes for Food and Drug Control, Beijing, China

**Keywords:** aryl hydrocarbon receptor, DNA damage, endoplasmic reticulum stress, lipid metabolism disorder, liver injury

## Abstract

The aryl hydrocarbon receptor (AhR) is a key regulatory factor that enables the organism to sense and integrate environmental and metabolic signals. Its biological function exhibits a universal “dual nature,” a feature that has been relatively well studied in organs such as the skin, lungs, and intestines. However, in the pathological processes of the liver—the central metabolic organ—the specific molecular mechanisms and regulatory networks that determine the functional orientation of AhR (whether protective or detrimental) remain a frontier and highly controversial area of research. This review aims to critically synthesize existing evidence, elucidating how AhR influences the progression of liver injury by regulating apoptosis, stress-induced damage, metabolic homeostasis, autophagy, fibrosis, and tumor development. It further explores the causes of its functional heterogeneity, such as ligand specificity and tissue microenvironment. By analyzing current controversies and cognitive gaps in the field, this article seeks to provide a framework for clarifying the complex role of AhR in liver diseases and to guide future exploration of targeted intervention strategies.

## Graphical Abstract

**Figure:**
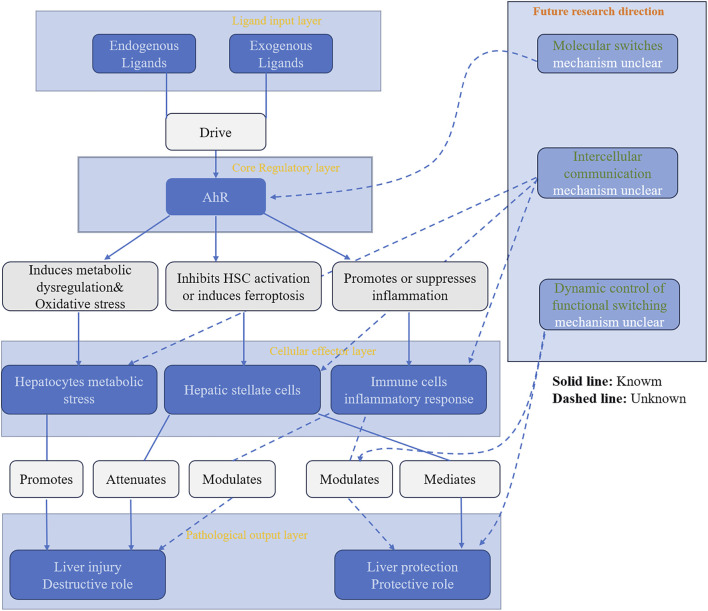

## 1 Introduction

The aryl hydrocarbon receptor (AhR) is a ligand-activated transcription factor originally identified for its role in mediating the harmful effects of environmental toxins such as dioxin. It has since been redefined as a central regulator capable of integrating dietary, microbial, and endogenous signals, playing a critical role in maintaining organismal homeostasis. Notably, the biological functions of AhR in barrier organs such as the intestine, lung, and skin have been extensively and deeply investigated. Its mechanisms of action in these sites—particularly in immune regulation, maintenance of epithelial barrier integrity, and host-environment interactions—are now relatively well understood (Zhang et al., 2022; Carambia and Schuran, 2021). By contrast, a systematic understanding of AhR’s role in liver pathology is still lacking, which is particularly intriguing given that the liver is a central metabolic organ constantly exposed to abundant gut-derived and endogenous ligands. Although AhR has been confirmed to be crucial for hepatic homeostasis—by maintaining immune tolerance and integrating signals to coordinate metabolism and immunity—its specific role in liver injury remains ambiguous and fraught with contradictions (Lahvis et al., 2000; Moreno-Marín et al., 2018; Walisser et al., 2005). It is important to note that the “dual” or “Janus-faced” nature of the AhR signaling pathway is a universal feature of its biological function and has been described in multiple tissues. However, within the unique metabolic and immune microenvironment of the liver, the specific molecular mechanisms and regulatory networks that determine its functional orientation (i.e., whether it exerts protective or damaging effects) represent the most understudied and fragmented area of current research.

It is precisely this lack of mechanistic understanding that makes the “double-edged sword” characteristic exhibited by AhR in liver injury the core issue in this field. As summarized in Table 1, depending on ligand properties, the cellular microenvironment, and the specific disease context, identical AhR signaling can either drive oxidative stress, inflammation, and fibrogenesis, or conversely, mediate cytoprotective and anti-inflammatory effects. This functional duality not only renders its therapeutic potential difficult to define but also highlights fundamental gaps in our mechanistic understanding and the lack of a unified theoretical framework. At its core, the molecular determinants—the precise “switches” that dictate the functional outcome of AhR signaling in liver injury—remain a critical and unresolved question demanding systematic investigation.

**TABLE 1:** Summary of key factors and evidence governing the functional orientation of the AhR in liver injury.

Class	Name	Source	Model	AhR KO	Impact on liver homeostasis
AhR agonist	TCDD	Exogenous	Mice	Yes	Attenuates liver fibrosis by inhibiting HSC activation (Yan et al., 2019)
			Mice HepG2	Yes	Promotes liver fibrosis development by inducing EMT via the AhR/Snail2 signaling pathway (Pierre et al., 2014)
			Mice Hepa1c2c7	Yes	Induces mitochondrial dysfunction by downregulating mitochondrial proteins and reducing oxygen consumption rate (Oh et al., 2025) Promotes hepatic lipid accumulation and inflammatory cell infiltration by inducing the expression and secretion of PAI-1 (Oh et al., 2025)
			Rat	Yes	Recruits neutrophils to the liver and promotes tissue inflammation by activating the ALOX5/LTB4 pathway (Takeda et al., 2017)
			Mice Rat	No	Promotes liver disease progression by regulating lncRNAs that modulate lipid metabolism, inflammatory response, extracellular matrix deposition, cell proliferation, apoptosis, or other signaling pathway (Post et al., 2025)
			Hepa1c2c7	No	Induces mitochondrial dysfunction by inhibiting the interaction between AhR and the ATP5α1 subunit, leading to mitochondrial membrane hyperpolarization (Tappenden et al., 2011)
			PMH PRH	No	Promotes lipid deposition by inducing SLC13A5 gene expression, increasing citrate uptake and *de novo* lipogenesis in hepatocytes (Neuschäfer-Rube et al., 2015)
			Huh-7	Yes	Increases lipid accumulation and HCV viral assembly via AhR-CYP1A1/1B1 (Ohashi et al., 2018)
			Mice	No	Exacerbates bile duct ligation-induced liver injury by regulating genes such as CYP1A1/1A2, TNF-α/IL-1β, and those involved in bile acid synthesis and transport (Ozeki et al., 2011)
			HepG2 PHH	Yes	Alleviates cholestasis, oxidative stress, and inflammation-induced liver injury by upregulating MRP4 expression (Xu et al., 2010)
			Mice	Yes	Induces mitochondrial oxidative stress by increasing glutathione (GSH) levels in hepatocyte mitochondria, leading to cytoplasmic and mitochondrial DNA damage (Shen et al., 2005)
	ITE	Host metabolism	Mice	Yes	Attenuates liver fibrosis by inhibiting HSC activation (Zhang et al., 2022)
				No	Aggravates APAP-induced hepatotoxicity by upregulating CYP1A2 and inducing neutrophil infiltration (Schuran et al., 2021)
	OTA	Exogenous	Mice HepG2	No Yes	Induces hepatocyte oxidative stress, DNA damage, and apoptosis by promoting the generation of toxic intermediates, while also affecting cellular detoxification capacity (Shin et al., 2019)
	Chrysene	Exogenous	Mice	Yes	Induces DNA damage through oxidative stress (Tao et al., 2021)
	BbF	Exogenous	Mice	No	Induces liver injury by triggering oxidative stress, lipid metabolism disorders, and inflammatory responses (Liu et al., 2025)
	BaP	Exogenous	HepG2	No	Induces intracellular lipid deposition in hepatocytes (Zhang et al., 2025)
			PMH PRH	No	Promotes lipid deposition by inducing SLC13A5 gene expression, increasing citrate uptake and *de novo* lipogenesis in hepatocytes (Neuschäfer-Rube et al., 2015)
			Hepa 1c1c7	Yes	Induces oxidative stress (Elbekai et al., 2004)
	ICA	Host metabolism	Mice	Yes	Suppresses excessive T cell activation and alleviates immune-mediated hepatitis by activating AhR on T cells, upregulating Pik3ip1 expression, and inhibiting the PI3K/Akt/mTOR signaling pathway (Li et al., 2025)
​	High-fat diet/FFA	Diet	Mice HepG2	Yes	Promotes hepatic lipid synthesis and accumulation by enhancing miR-132 expression, leading to downregulation of SIRT1 and subsequent upregulation of lipogenic genes (Tao et al., 2025)
​	lenvatinib	Drug	HuH7 Hep3B	Yes	Confers lenvatinib resistance in HCC cells by promoting AREG expression, thereby activating the EGFR-ERK1/2-CyclinD1 signaling pathway (Hu et al., 2025)
​	YH439	Synthetic	mHSCs	No	Alleviates mouse liver fibrosis by selectively promoting ferroptosis in mHSCs without inducing ferroptosis in hepatocytes (Liu, 2024)
​	Kyn	Host metabolism	Mice AML12	Yes	Maintains liver mitochondrial homeostasis by regulating BNIP3 expression. AhR deficiency leads to mitochondrial dysfunction, increased ROS production, and suppressed mitophagy (Heo et al., 2023)
​	TCDF	Exogenous	Mice	No	Induces hepatic lipid accumulation by upregulating Sptlc2, resulting in ceramide accumulation and promotion of adipogenesis (Liu et al., 2021)
​	Cl-PAHs	Exogenous	H4IIE	No	Induces DNA damage in a dose-dependent manner (Tao et al., 2025)
​	HCV	Virus	Huh-7	Yes	Increases lipid accumulation and HCV viral assembly via AhR-CYP1A1 (Ohashi et al., 2018)
​	HCY	Host metabolism	Mice PMH	No	Induces fatty liver by activating the LXA4-AhR-CD36 signaling pathway, increasing lipid uptake (Yao et al., 2016)
​	3-MC	Exogenous	PMH PRH	No	Promotes lipid deposition by inducing SLC13A5 gene expression, increasing citrate uptake and *de novo* lipogenesis in hepatocytes (Neuschäfer-Rube et al., 2015)
​			HepG2	Yes	Alleviates cholestasis, oxidative stress, and inflammation-induced liver injury by upregulating MRP4 expression (Xu et al., 2010)
​			Hepa 1c1c7	Yes	Induces oxidative stress (Elbekai et al., 2004)
​	HCB	Exogenous	Rat HepG2	No	Promotes the formation of preneoplastic lesions and cell proliferation, and modulates cell cycle alterations in rat liver by inducing the AhR/ERK1/2 signaling pathway (de Tomaso Portaz et al., 2015)
​	DIM	Host metabolism	HSC-T6 HSC	No	Attenuates liver fibrosis by downregulating miR-21 expression, inhibiting the TGF-β signaling pathway, and suppressing HSC activation (Zhang et al., 2013)
​	—	—	Mice	No	Compared with AhR^+/+^ mice, AhR^−/−^ mice exhibited higher tumor incidence, increased tumor number, larger tumor size, and enhanced inflammatory response after DEN treatment (Fan et al., 2010)
​	OPZ	Drug	HepG2 PHH	Yes	Alleviates cholestasis, oxidative stress, and inflammation-induced liver injury by upregulating MRP4 expression (Xu et al., 2010)
​	4-PBA	Synthetic	HepG2	Yes	Alleviates cholestasis, oxidative stress, and inflammation-induced liver injury by upregulating MRP4 expression (Xu et al., 2010)
​	B[a]P-7,8-dione	Exogenous	Hepa1c1c7 Hepa1c1c4 Hepa1c1c12	No	Leads to DNA single-strand breaks by binding AhR, translocating to the nucleus, and generating ROS (Park et al., 2009)
​	“X”	Endogenous	Hepa-1c1c7	No	Induces cell cycle arrest, particularly in G1 phase, by promoting AhR-RB complex formation and inhibiting E2F transcription (Puga et al., 2002)
​	β-NF	Exogenous	Hepa1c1c7	Yes	Induces oxidative stress (Elbekai et al., 2004)
AhR antagonist	HBU651	Synthetic	Mice Hepa1c2c7	Yes	Reduces lipid accumulation by significantly suppressing TCDD-induced PAI-1 expression (Oh et al., 2025)
	CH-223191	Synthetic	Mice AML12	Yes	Induces mitochondrial dysfunction by reducing mitochondrial respiration rate, membrane potential, and utilization of tricarboxylic acid cycle substrates (Heo et al., 2023)
			Huh-7	Yes	Reduces lipid accumulation and HCV viral assembly via the AhR-CYP1A1 axis (Ohashi et al., 2018)
			Mice PMH	Yes	Alleviates HHcy-induced hepatic steatosis by inhibiting the AHR/CD36 pathway (Yao et al., 2016)
			PMH PRH	No	Inhibits citrate uptake and *de novo* lipogenesis in hepatocytes by suppressing the AhR/SLC13A5 pathway (Neuschäfer-Rube et al., 2015)
	Flutamide	Drug	Huh-7	Yes	Reduces lipid accumulation and HCV viral assembly via the AhR-CYP1A1 axis (Ohashi et al., 2018)
	—	—	Mice PMH	Yes	Disrupts bile acid homeostasis, inducing cholestasis and liver necrosis, by upregulating the bile acid transporter ABCC4 and inhibiting FXR signaling (Gao et al., 2016)
	4,7-OP	Synthetic	Rat HepG2	No	Inhibits the formation of preneoplastic lesions and cell proliferation, and modulates cell cycle alterations in rat liver by suppressing the AhR/ERK1/2 signaling pathway (de Tomaso Portaz et al., 2015)
	Resveratrol	Exogenous	HepG2	No	Exerts anti-cancer and detoxification effects by indirectly inhibiting TCDD-induced binding of AhR and ARNT to enhancer regions of CYP1A1 and CYP1B1 genes, thereby suppressing RNA polymerase II binding at promoter regions (Beedanagari et al., 2009)

HSC, hepatic stellate cells; EMT, epithelial-mesenchymal transition; HC, hepatocytes; KC, kupffer cells; OTA, Ochratoxin A; FFA, free fatty acids; APAP, acetaminophen; HHcy, Hyperhomocysteinemia; PMH, primary mice hepatocytes; PRH, primary rat hepatocytes; 3-MC, 3-Methylcholanthrene; PHH, primary human hepatocytes; “X” denotes an endogenous AhR activator.

Accordingly, this review aims to critically organize and integrate the current fragmented research on AhR and liver injury (Table 1). By focusing on the conflicting evidence regarding the role of AhR in hepatocyte apoptosis, stress response, metabolic dysregulation, fibrosis, and cancer development, we seek to outline key knowledge gaps in the field. We anticipate that this work will establish a logical framework to support future research in more rationally exploring novel AhR-targeted intervention strategies for liver diseases.

## 2 Role and molecular mechanisms of the aryl hydrocarbon receptor in liver injury

### 2.1 Aryl hydrocarbon receptor and hepatocyte apoptosis

#### 2.1.1 Aryl hydrocarbon receptor and mitochondrial damage

Mitochondria serve not only as the cellular “powerhouse,” responsible for generating approximately 80% of the cell’s energy, but also play crucial roles in maintaining electrolyte homeostasis, regulating reactive oxygen species (ROS) production, and mediating cellular signaling. The structural and functional integrity of mitochondria is essential for hepatocyte survival.

The activation of the AhR has been identified as a significant mechanism that induces mitochondrial dysfunction and subsequently promotes hepatocyte apoptosis. In terms of oxidative stress, Shertzer et al. demonstrated that TCDD alters the redox state of mitochondrial glutathione (GSH) in an AhR-dependent manner, leading to excessive production of mitochondrial ROS (mtROS) and subsequent oxidative damage to mitochondrial DNA (mtDNA) (Shen et al., 2005). Similarly, in hepatocyte models treated with polycyclic aromatic hydrocarbons such as benzo[a]pyrene (B[a]P), AhR activation also relies on its transcriptional activity to induce oxidative stress and lipid peroxidation (Elbekai et al., 2004). This sustained oxidative stress is a key driver of hepatocyte apoptosis. Regarding mitochondrial membrane potential and energy metabolism, LaPres et al. discovered that AhR activation disrupts the mitochondrial membrane potential (MMP) through multiple pathways (Tappenden et al., 2011). On one hand, AhR agonists can induce abnormal opening of the mitochondrial permeability transition pore (mPTP), leading to MMP collapse and a sharp decrease in ATP synthesis, triggering a cellular energy crisis. On the other hand, AhR can also participate in regulation through non-transcriptional mechanisms, such as direct interaction with the ATP synthase subunit ATP5α1, causing mitochondrial hyperpolarization following TCDD exposure (Tappenden et al., 2011). While this hyperpolarization may represent a compensatory response to maintain energy homeostasis, its persistence indicates severe functional dysregulation. Furthermore, AhR activation inhibits mitochondrial respiratory function and substrate utilization capacity, downregulating the expression of energy metabolism-related genes and fundamentally impairing cellular energy supply (Oh et al., 2025; Liu et al., 2025; Heo et al., 2023).

Mitophagy, the core quality control mechanism for clearing damaged mitochondria, is also intricately regulated by AhR. Kim et al. revealed the dual role of AhR in this process: under physiological conditions, AhR promotes the clearance of damaged mitochondria and maintains mitochondrial homeostasis by transcriptionally regulating the autophagy receptor BNIP3 (Heo et al., 2023). However, persistent overactivation or dysregulation of AhR signaling can disrupt mitophagy. Once this quality control mechanism fails, damaged mitochondria accumulate, mtROS levels rise further, creating a vicious cycle of oxidative stress and inflammatory responses that ultimately accelerates hepatocyte apoptosis.

Although current studies have preliminarily constructed a potential framework in which AhR activation drives hepatocyte apoptosis by inducing mitochondrial oxidative stress, disrupting energy homeostasis, and interfering with autophagy, it must be acknowledged that this framework is still largely based on limited correlative research, reflecting significant knowledge gaps in the field. The core deficiencies currently lie in: the existence of a “mechanistic disconnect” in understanding the downstream effectors through which AhR regulates mitochondrial function (such as its direct transcriptional regulation of nuclear genes encoding electron transport chain components or the pathological significance of its interactions with mitochondrial proteins); and the general neglect of the potential cell-specific functions of AhR signaling across different liver cell populations (e.g., hepatocytes, stellate cells). Therefore, future research urgently needs to precisely identify the direct mitochondrial targets of AhR using liver-specific genetic manipulation models and validate these findings in clinically relevant systems such as human liver tissue and organoids. This will advance the study of the AhR-mitochondria axis from phenomenological association to causal mechanism, providing new perspectives for understanding and intervening in environmental and metabolic liver diseases.

#### 2.1.2 Aryl hydrocarbon receptor and DNA damage

DNA damage, as a key event affecting genomic stability, plays a central role in hepatocyte mutation, apoptosis, and carcinogenesis. The AhR, a crucial sensor of environmental signals, participates in the DNA damage process through multiple mechanisms upon activation: By directly upregulating the expression of metabolizing enzymes such as cytochrome P450s (CYP1A1, CYP1B1) and aldo-keto reductases (AKRs), AhR promotes the metabolic activation of procarcinogens like polycyclic aromatic hydrocarbons (PAHs) (Huang et al., 2018). The resulting electrophilic intermediates can directly form covalent adducts with DNA, a process accompanied by substantial generation of ROS, leading to DNA strand breaks and oxidative base modifications (Shen et al., 2005; Park et al., 2009; Gao et al., 2016).

However, the role of AhR in DNA damage exhibits significant duality. Studies by Puga et al. demonstrated that AhR deficiency, paradoxically, leads to increased accumulation of DNA damage and promotes tumorigenesis, suggesting a potential protective role in maintaining genomic stability under specific conditions (Fan et al., 2010). This functional contradiction underscores the complex role of AhR in the DNA damage process. Whether it acts as a “driver” or a “guardian” likely depends on the interplay between specific environmental exposures, genetic background, and pathological status. Resolving this critical scientific question requires further in-depth investigation.

#### 2.1.3 Aryl hydrocarbon receptor-induced cell cycle arrest

Cell cycle arrest refers to the phenomenon where the cell proliferation process is interrupted at a specific stage, preventing cells from completing division. This process can be triggered by various factors, including DNA damage, activation of cell cycle checkpoints, and dysregulation of intracellular signaling pathway.

As a key transcriptional regulator of the cell cycle, the AhR exhibits dual regulatory characteristics in response to genotoxic stress. In a protective response, ligand-mediated activation of AhR can directly interact with the retinoblastoma protein (RB), inhibit E2F-dependent transcriptional activity, and induce G1 phase cell cycle arrest, creating a critical time window for DNA repair (Puga et al., 2002). However, under pathological conditions, the AhR signaling pathway demonstrates an opposite, pro-proliferative effect. Hexachlorobenzene activates the ERK1/2 signaling pathway in an AhR-dependent manner, upregulates the expression of the cell cycle protein Cyclin D1, and promotes the proliferation of hepatocellular carcinoma cells and hepatic preneoplastic lesions (de Tomaso Portaz et al., 2015). Similarly, in hepatocellular carcinoma, the development of lenvatinib resistance is closely associated with the aberrant activation of the AHR-AREG-EGFR-ERK1/2-Cyclin D1 signaling axis. Here, AhR drives cell cycle progression by promoting the expression of the autocrine factor AREG (Hu et al., 2025). It is noteworthy that the absence of AhR also leads to dysregulation of cell cycle control. Studies have shown that AhR knockout mice exhibit a higher incidence of liver tumors induced by the chemical carcinogen DEN. The mechanism involves the accumulation of DNA damage, upregulation of proliferation markers, and failure of cell cycle checkpoints, confirming that AhR, under physiological conditions, functions as a tumor suppressor by restraining abnormal cell cycle progression (Fan et al., 2010).

In summary, AhR acts as an environmental response “double-edged sword” in cell cycle regulation: it serves as a protective “brake” under genotoxic stress, inducing cycle arrest to maintain genomic stability, and yet can act as a proliferative “accelerator” in disease pathogenesis, promoting progression via pathways such as ERK/Cyclin D1. The molecular mechanisms underlying this functional switch and its specific regulation in liver diseases remain important directions for future research.

### 2.2 Aryl hydrocarbon receptor and hepatocyte stress injury

#### 2.2.1 Aryl hydrocarbon receptor and oxidative stress

Oxidative stress refers to a phenomenon where the redox balance within cells or tissues is disrupted, leading to abnormally elevated levels of ROS and subsequent cellular damage. Research indicates that the activation of the AhR drives the initiation and progression of hepatic oxidative stress through multiple synergistic mechanisms. Through direct mechanisms level, AhR ligands such as TCDD can induce severe mitochondrial dysfunction, significantly stimulating the overproduction of mtROS by altering the mitochondrial glutathione redox state, and directly causing specific oxidative damage to mitochondrial DNA (Shen et al., 2005). Concurrently, polycyclic aromatic hydrocarbon AhR ligands, including B[a]P, 3-MC, and β-NF, also induce significant oxidative stress and lipid peroxidation in hepatocytes via AhR-dependent pathways (Elbekai et al., 2004). Indirectly, AhR triggers the substantial generation of ROS as a byproduct during the metabolic activation of xenobiotics, by transcriptionally regulating the expression of metabolizing enzymes such as cytochrome P450s (e.g., CYP1A1) and aldo-keto reductases. This subsequently leads to secondary damage, including DNA strand breaks and oxidative base modifications (Liu et al., 2025; Park et al., 2009). The scenario is particularly complex due to the dynamic interaction between the AhR signaling network and the intracellular antioxidant system. On one hand, in models of toxin exposure such as to OTA, AhR can activate the Nrf2 signaling pathway, thereby inducing the expression of Phase II antioxidant enzymes, including HO-1 and GCLC, forming an adaptive protective mechanism (Shin et al., 2019). On the other hand, in hepatic stellate cells, the AhR agonist YH439 promotes massive glutathione (GSH) efflux by upregulating Mrp1 protein, directly depleting intracellular reserves of this crucial antioxidant. This creates a pro-oxidant environment and induces ferroptosis (Liu, 2024). A similar phenomenon of GSH depletion was also confirmed in a phenanthrene-induced hepatotoxicity model (Tao et al., 2021).

However, significant gaps remain in the current understanding of AhR’s regulation of oxidative stress: the specific regulatory networks of AhR signaling in different liver cell types are not yet clear, and the molecular switches that determine the shift of AhR towards a pro-oxidant or antioxidant balance under different pathological contexts remain poorly understood.

#### 2.2.2 Aryl hydrocarbon receptor and endoplasmic reticulum stress

Endoplasmic reticulum stress (ERS) is a crucial cellular response triggered by impaired ER function and the accumulation of unfolded/misfolded proteins. The ensuing unfolded protein response (UPR) aims to restore proteostasis; however, sustained or severe ERS ultimately induces apoptosis. Current research indicates a well-established link between aryl hydrocarbon receptor (AhR) activation and ERS in non-hepatic tissues such as the lung and intestine: AhR can induce ERS by promoting reactive oxygen species (ROS) generation, disrupting calcium ion homeostasis, and directly modulating key UPR signaling axes (e.g., PERK-eIF2α-ATF4). It may also mitigate its toxic effects by regulating autophagy (Wang et al., 2017; Guerrina et al., 2021; Kunitomi et al., 2021; Lai et al., 2014).

However, the existence and function of this important AhR-ERS regulatory axis in the liver—a central organ for metabolism and xenobiotic detoxification—currently remains a significant knowledge gap. Although the broad body of research covered in this review has confirmed that AhR can induce liver injury through various mechanisms—such as direct mitochondrial oxidative stress (Shen et al., 2005; Elbekai et al., 2004), metabolic activation leading to DNA damage (Park et al., 2009), and disruption of lipid metabolism (Neuschäfer-Rube et al., 2015; Liu et al., 2021)—none of these studies have addressed the critical scientific question of whether and how AhR participates in regulating endoplasmic reticulum homeostasis in hepatocytes.

#### 2.2.3 Aryl hydrocarbon receptor and inflammatory stress

Inflammatory stress refers to the stress response triggered by inflammatory stimuli in cells, involving the synthesis of inflammatory mediators, infiltration of inflammatory cells, and inflammation-related apoptosis and necrosis. Studies have demonstrated that AhR activation can drive hepatic inflammatory responses through direct regulation of immune cell function, induction of pro-inflammatory mediator production, and interaction with other injury-related signaling pathways (Pierre et al., 2014; Takeda et al., 2017; Ozeki et al., 2011; Schuran et al., 2021; Liu et al., 2025; Li et al., 2025).

In terms of initiation mechanisms, AhR activation can directly mediate neutrophil infiltration into the liver. Research by the Yamada team revealed that TCDD, in an AhR-dependent manner, significantly promotes the biosynthesis of the potent chemoattractant leukotriene B4 (LTB4) in the liver, leading to marked neutrophil infiltration. Notably, this inflammatory process and the accompanying liver injury were effectively blocked in BLT1 (the LTB4 receptor) knockout mice, confirming the central role of LTB4 in this pathway (Takeda et al., 2017). Concurrently, Coumoul et al. highlighted the significant role of direct AhR transcriptional regulation in inflammation: TCDD, via direct binding and activation of the Snai2 gene promoter by AhR, induces epithelial-mesenchymal transition (EMT). This process is closely associated with the upregulation of inflammatory factors such as IL-1β and TNF-α, collectively promoting the progression of liver fibrosis (Pierre et al., 2014).

Regarding amplification mechanisms, AhR acts as a bridge connecting oxidative stress and inflammatory responses. Studies by Liu et al. showed that polycyclic aromatic hydrocarbons like Benzo[b]fluoranthene (BbF), through activating the AhR pathway, not only lead to a significant increase in pro-inflammatory factors such as TNF-α, IL-1β, IL-6, and IL-8 but are also accompanied by oxidative stress and lipid metabolism disorders, creating a vicious cycle where inflammation and metabolic abnormalities mutually exacerbate each other (Liu et al., 2025). Similarly, the Carambia team, in an acetaminophen (APAP)-induced liver injury model, found that treatment with the AhR agonist ITE not only upregulated the toxic metabolic enzyme CYP1A2 but also triggered significant neutrophil infiltration and excessive production of inflammatory cytokines including IL-6, TNF-α, and IL-1β, collectively exacerbating liver injury (Schuran et al., 2021). Further supporting this, Makishima et al., in a cholestasis model, demonstrated that TCDD pretreatment significantly enhanced the production of inflammatory cytokines such as TNF and IL-1B in the plasma of bile duct ligated (BDL) mice, indicating that AhR signaling can markedly worsen cholestasis-associated inflammatory responses (Ozeki et al., 2011).

It is noteworthy that AhR exhibits a significant context-dependent dual role in inflammatory regulation. In an autoimmune hepatitis (AIH) model, the Ma team discovered that the gut microbial metabolite indole-3-carboxaldehyde (ICA), by activating AhR on T cells, upregulates Pik3ip1 expression, inhibits the PI3K/Akt/mTOR signaling pathway, thereby suppressing excessive T cell activation and IFN-γ production while promoting the expansion of regulatory T cells (Tregs). Ultimately, this alleviates immune-mediated inflammatory liver injury (Li et al., 2025). This finding reveals the anti-inflammatory therapeutic potential of specific AhR ligands in immune-mediated liver diseases.

In summary, AhR plays a complex and critical role in the hepatic inflammatory network by regulating various inflammatory mediators and immune cell functions. Future research needs to further elucidate the molecular switches governing the transition between pro-inflammatory and anti-inflammatory effects mediated by different AhR ligands, as well as the specific mechanisms by which AhR signaling regulates inflammation in different liver cell types. This will provide a theoretical foundation for developing AhR-targeted strategies to modulate liver inflammation.

### 2.3 Aryl hydrocarbon receptor and hepatocyte metabolic disorders

#### 2.3.1 Aryl hydrocarbon receptor and cholestasis

Cholestasis is a pathological condition caused by impaired synthesis, secretion, or excretion of bile acids (BAs) in the liver. Its long-term persistence can lead to liver fibrosis, cholangitis, and even progression to cirrhosis (Ozeki et al., 2011).

The role of the AhR in the cholestatic process exhibits significant complexity and duality, with its functional output highly dependent on ligand properties, disease stage, and cellular context. Studies demonstrate that the anti-androgen drug flutamide (FLU), acting as an AhR agonist, triggers a significant increase in serum and hepatic bile acid levels accompanied by hepatomegaly via AhR signaling activation. These toxic effects were completely absent in AhR knockout mice, confirming the central role of AhR in this process (Gao et al., 2016). Similarly, the environmental pollutant TCDD was found to exacerbate liver injury in a bile duct ligation model in an AhR-dependent manner, manifested by further elevated bile acid and bilirubin levels and more severe hepatic necrosis (Ozeki et al., 2011).

In stark contrast to these injurious effects, AhR also demonstrates clear protective functions under specific conditions. In a DDC-induced chronic cholestasis model, AhR overexpression significantly improved liver injury parameters, reduced serum total bilirubin and alkaline phosphatase levels, and alleviated hepatotoxicity by selectively reducing the accumulation of tauro-conjugated bile acids (Han et al., 2023). The mechanism underlying this protection may involve direct transcriptional regulation by AhR of transporters such as the multidrug resistance-associated protein 4 (MRP4), thereby enhancing hepatocyte capacity to excrete toxic substances (Xu et al., 2010). It is noteworthy that downstream effectors of AhR themselves exhibit functional divergence; for instance, CYP1A1 and CYP1A2 induced by TCDD were shown to be protective in Cyp1a1/1a2 double-knockout mice, revealing the complexity of the AhR signaling pathway (Ozeki et al., 2011).

The molecular basis for this functional duality remains to be fully elucidated. Interspecies research variations add complexity; for example, in the transparent carp model, bilirubin activated mouse AhR but failed to activate zebrafish AhR1a, suggesting potential species specificity in protective signaling mediated by endogenous ligands (Volz et al., 2008). A critical gap in current research lies in understanding the molecular switches that determine the functional orientation of AhR and its specific action networks within different liver cell types, such as hepatocytes and cholangiocytes.

#### 2.3.2 Aryl hydrocarbon receptor and lipid metabolism disorder

Lipid metabolism disorder refers to abnormalities in processes such as fatty acid synthesis, oxidation, and transport, leading to intracellular lipid accumulation or impaired utilization. The resulting lipid buildup can induce endoplasmic reticulum stress and inflammatory responses, thereby causing hepatocyte damage or even apoptosis.

Extensive studies have demonstrated that upon exposure to environmental pollutants (e.g., TCDD, BaP, HCB), the activation of the AhR significantly drives the initiation and progression of hepatic steatosis through a synergistic, multi-target network. The mechanisms primarily manifest at four levels: First, AhR induces direct transcriptional reprogramming, upregulating the expression of key lipogenic genes including SREBP-1c, FAS, ACC, and SCD1, and provides ample precursors for lipid synthesis by inducing the citrate transporter mINDY (SLC13A5) (Neuschäfer-Rube et al., 2015; Liu et al., 2021). Second, it enhances fatty acid uptake capacity via upregulation of CD36, while simultaneously inhibiting fatty acid β-oxidation by suppressing AMPK activity and inducing mitochondrial dysfunction (e.g., reduced oxygen consumption rate, increased ROS production), thereby disrupting lipid metabolic balance (Shen et al., 2005; Liu et al., 2025; Yao et al., 2016). Third, it activates the *de novo* ceramide synthesis pathway, exacerbating lipotoxic effects (Liu et al., 2021). Fourth, AhR-induced oxidative stress and inflammatory responses form a vicious cycle with lipid metabolism disorders, collectively driving disease progression (Shin et al., 2019; Liu et al., 2025).

It is particularly noteworthy that AhR’s regulation of metabolic hormones such as FGF21 exhibits a refined dose-dependent characteristic, which may represent a fine-tuning mechanism for switching between adaptive responses and toxic effects (Zhang et al., 2025). However, AhR’s role in lipid metabolism is not solely that of a “promoter.” Under specific conditions, such as when activated by β-naphthoflavone (β-NF) or when maintaining its endogenous activity, AhR can basally suppress the expression of fatty acid synthesis genes in a non-DRE-dependent manner, functioning as a metabolic “brake” (Tanos et al., 2012). This functional paradox fully illustrates the highly context-dependent nature of the AhR signaling pathway, whose ultimate effect is profoundly influenced by the combined factors of ligand properties, cell type, and the overall pathophysiological background.

Although some progress has been made in understanding the role of AhR in hepatic lipid metabolism, significant knowledge gaps remain in several key areas. For instance, the specific mechanisms of the non-canonical (non-DRE-dependent) AhR pathway in lipid metabolism are poorly understood. Why do different AhR ligands (e.g., TCDD vs. β-NF), or even different concentrations of the same ligand (e.g., the bidirectional regulation of FGF21 by BaP), lead to markedly different metabolic outcomes? Future research should systematically compare the effects of different agonists, antagonists, and selective AhR modulators (sAhRMs), aiming to screen or design targeted AhR therapies for metabolic diseases that can harness its benefits while mitigating its detrimental effects.

#### 2.3.3 Aryl hydrocarbon receptor and abnormal expression of hepatocyte metabolic enzymes

The AhR, functioning as a xenobiotic sensor, is most classically known as the primary transcriptional regulator of the cytochrome P450 (CYP) enzyme system, particularly the CYP1 family, thereby dominating Phase I metabolism of numerous foreign compounds in the liver. Its canonical pathway initiates with ligand binding, followed by nuclear translocation of AhR and heterodimerization with ARNT. This complex recognizes and binds to the xenobiotic response elements (XRE/DRE) in the promoter regions of target genes, subsequently initiating transcription (Gao et al., 2016). Extensive studies confirm that CYP1A1 is its most sensitive and representative target gene. Its mRNA and protein expression, along with ethoxyresorufin-O-deethylase (EROD) activity, are strongly induced by various AhR ligands such as TCDD, BaP, 3-methylcholanthrene, and flutamide, making it a frequently used biomarker for AhR activation (Pierre et al., 2014; Post et al., 2025; Elbekai et al., 2004; Beedanagari et al., 2009). Concurrently, CYP1A2 and CYP1B1 are co-regulated, collectively forming the core network for AhR-mediated metabolic activation (Ozeki et al., 2011; Schuran et al., 2021; Tao et al., 2021). Notably, AhR’s regulatory scope extends far beyond CYP enzymes; it can also induce arachidonate 5-lipoxygenase (ALOX5), thereby altering eicosanoid metabolic balance and promoting leukotriene B4 synthesis, tightly linking the xenobiotic response to inflammatory processes (Takeda et al., 2017).

Beyond presiding over Phase I metabolism, AhR activation coordinately initiates a broad “detoxification program” involving Phase II metabolizing enzymes and efflux transporters to facilitate the clearance of metabolites. This includes the regulation of enzymes such as NAD(P)H quinone dehydrogenase 1 (NQO1), glutathione S-transferases (GSTs), UDP-glucuronosyltransferases (UGTs), and sulfotransferases (SULTs) (Xu et al., 2010; Tao et al., 2021) Crucially, AhR, alongside nuclear factor erythroid 2-related factor 2 (Nrf2), has been identified as a key transcriptional regulator of multidrug resistance-associated protein 4 (MRP4/ABCC4). It can directly bind to its promoter region and induce its expression under stress conditions such as cholestasis, highlighting AhR’s vital role in maintaining bile acid homeostasis (Xu et al., 2010; Gao et al., 2016). This comprehensive metabolic program, encompassing activation, detoxification, and efflux, holds profound pathophysiological significance: it is the core process by which many procarcinogens (e.g., Benzo[a]pyrene) are metabolically activated into ultimate carcinogens (Zhang et al., 2025; Park et al., 2009); it is a key determinant in susceptibility to drug-induced liver injury (DILI) (e.g., by inducing CYP1A2 to exacerbate acetaminophen hepatotoxicity) (Schuran et al., 2021); and the reactive oxygen species (ROS) generated during its metabolic processes are a significant source of oxidative stress induced by AhR ligands (Elbekai et al., 2004).

Although the network of AhR-regulated metabolic enzymes is now well-characterized, current research has primarily focused on gene regulation via the canonical, DRE-dependent pathway. Future investigations need to delve deeper into the roles of non-canonical pathways and post-transcriptional regulation in shaping the metabolic enzyme expression profile. Furthermore, the liver is a complex organ composed of multiple cell types. Utilizing cell-specific knockout models to precisely dissect the cell-specific roles of AhR—in hepatocytes, cholangiocytes, hepatic stellate cells, and Kupffer cells—in regulating metabolic enzymes, along with its role in intercellular communication, will significantly advance our understanding of the liver’s overall detoxification capacity.

### 2.4 Aryl hydrocarbon receptor and hepatocyte autophagy

Autophagy is a strictly regulated cellular degradation and recycling process that maintains intracellular homeostasis and biological balance by clearing damaged or aged organelles, proteins, and other cellular components. In hepatocytes, autophagy plays a key regulatory role in various physiological and pathological conditions, particularly under stresses such as nutrient deficiency, oxidative stress, and drug toxicity. Hepatocyte autophagy can be activated to maintain cell survival and mitigate damage. The precise regulation of hepatocyte autophagy involves multiple signaling pathways and key proteins, with the mammalian target of rapamycin (mTOR) pathway and AMP-activated protein kinase (AMPK) being the core regulators. The mTOR pathway, as a major negative regulator of autophagy, is inhibited under low cellular energy conditions, thereby promoting autophagy initiation, while AMPK activation directly stimulates the autophagic process. Imbalances in autophagy regulation, whether excessive activation or suppression, can impair liver structure and function.

Studies have shown that BNIP3 can induce cellular autophagy, and its degradation pathway involves both autophagy and the ubiquitin-proteasome system, with this process being regulated by AhR. Specifically, when AhR expression levels are low, BNIP3 expression is significantly upregulated, suggesting that BNIP3 plays a key role in AhR-mediated enhancement of autophagy (Tsai et al., 2017). Additionally, AhR itself plays an important role in the ubiquitin-proteasome degradation system and may further influence autophagy levels through protein-protein interactions with BNIP3. Notably, some studies have also found that AhR knockdown reduces the expression of autophagy-related factors, and AhR-regulated autophagy can affect inflammatory responses in human keratinocytes via the p65NF-κB/p38MAPK signaling pathway (Kim et al., 2020).

However, current research on the specific regulatory mechanisms of AhR in hepatocyte autophagy remains insufficient, and future studies are urgently needed to explore its role in maintaining liver homeostasis and the development of liver diseases.

### 2.5 Aryl hydrocarbon receptor and hepatocyte fibrosis

The core pathological process of hepatic fibrosis is the activation of HSCs and the excessive deposition of extracellular matrix. In recent years, the role of the AhR in this process has become increasingly clear, yet its function exhibits significant cell specificity and ligand dependency, constituting a complex dual regulatory network.

Within HSCs, AhR has been identified as a crucial endogenous anti-fibrotic brake. Research clearly demonstrates that AhR is highly expressed in quiescent HSCs, and its signaling pathway is vital for suppressing HSC activation. For instance, specific knockout of AhR in HSCs is sufficient to trigger spontaneous liver fibrosis in mice and enhance their susceptibility to chemically induced fibrosis (Yan et al., 2019). The protective mechanism involves interference with classic pro-fibrotic signaling pathways, such as inhibiting TGFβ-induced gene expression through interaction with Smad3 (Yan et al., 2019). More novelly, studies have found that non-toxic AhR ligands can induce ferroptosis in HSCs, thereby clearing activated HSCs and achieving an anti-fibrotic effect. This process depends on AhR upregulating multidrug resistance-associated protein 1 (Mrp1) and the subsequent triggering of glutathione depletion and lipid peroxidation (Liu, 2024). Furthermore, the natural product 3,3′-Diindolylmethane (DIM) also exerts therapeutic effects via an AhR-related pathway by downregulating the pro-fibrotic factor miR-21 (Zhang et al., 2013). These findings collectively establish that activating the AhR signal within HSCs is a highly promising anti-fibrotic strategy.

However, this clear picture becomes complicated when contrasted with environmental exposure studies. Despite the protective role of AhR within HSCs, exposure to potent environmental AhR ligands (such as the dioxin TCDD) is often reported to promote liver fibrosis (Pierre et al., 2014). This paradox suggests that the pro-fibrotic effect under systemic exposure may be an indirect consequence. The mechanism likely stems from the excessive activation of AhR in other liver cells (such as hepatocytes and immune cells), which creates a pro-fibrotic, injury-prone microenvironment that continuously drives HSC activation through mechanisms like inducing hepatocyte necrosis, robust inflammatory responses, and epithelial-mesenchymal transition (EMT). This indirect drive can overwhelm or even reverse the direct protective effect of AhR within HSCs (Pierre et al., 2014; Ozeki et al., 2011). Additionally, the function of AhR as a liver tumor suppressor links it to the fibrosis-hepatocarcinoma axis, as its loss leading to spontaneous liver injury and hyperplasia itself constitutes a strong pro-fibrotic stimulus (Fan et al., 2010).

Most current research on AhR in hepatic fibrosis focuses on preventing fibrosis progression. A question with greater clinical relevance is: Can AhR activation drive the reversal of established fibrosis? Investigating the role of AhR in regulating the reversion of HSCs from an activated state towards senescence, apoptosis, or inactivation would significantly expand its therapeutic prospects. Furthermore, current conclusions are largely derived from mouse models. There is an urgent need to validate the universality of these core mechanisms (such as the AhR-Smad3 interaction and susceptibility to induced ferroptosis) in human fibrotic liver tissues and primary HSCs. This validation represents the final hurdle for the clinical translation of anti-fibrotic therapies targeting AhR.

### 2.6 Aryl hydrocarbon receptor and hepatocellular carcinoma

The AhR exhibits a complex and context-dependent dual role in the development and progression of hepatocellular carcinoma (HCC), with its function varying significantly across different tumor stages. During the initiation stage of HCC, AhR primarily acts as a tumor suppressor. For instance, Puga and colleagues, using genetic models, demonstrated that Ahr-knockout mice exhibit significantly increased HCC incidence, tumor multiplicity, and size upon induction by chemical carcinogens. Mechanistic studies indicate that AhR exerts its tumor-suppressive functions by maintaining genomic stability, regulating cell cycle progression, and suppressing tumor-associated inflammatory responses (Fan et al., 2010). However, as the tumor progresses, the function of AhR undergoes a significant shift from tumor suppression to promoting malignancy. Particularly in the treatment resistance phase, the AhR signaling pathway can be co-opted by tumor cells to foster disease progression. For example, An et al. found that in lenvatinib-resistant HCC cells, AhR is aberrantly activated and drives a sustained pro-proliferative signaling pathway (AREG → EGFR → ERK1/2 → Cyclin D1) by directly upregulating the autocrine factor amphiregulin (AREG), ultimately leading to treatment failure (Hu et al., 2025). Furthermore, the canonical function of AhR as an environmental sensor intimately links it to the environmental etiology of liver cancer. AhR-regulated metabolizing enzymes, such as CYP1A1, are key players in the hepatic conversion of various environmental carcinogens (e.g., Benzo[a]pyrene) into genotoxic products (Elbekai et al., 2004; Tao et al., 2025).

This paradox—the shift from tumor suppression to oncogenesis—highlights a critical gap in current research and points toward future investigative directions: A deeper exploration of the molecular mechanisms governing this functional switch in AhR is crucial. This includes identifying key co-activators/co-repressors and post-transcriptional modifications that regulate AhR’s transcriptional activity and target gene selectivity at different stages of HCC. Utilizing advanced models such as conditional knockout and lineage tracing to thoroughly analyze, *in vivo*, the interactions between AhR function and pivotal signaling pathways like PTEN/PI3K/AKT and p53 will help systematically elucidate the molecular pathways underlying this functional transition. This understanding will provide a theoretical foundation for developing novel therapeutic strategies.

### 2.7 Aryl hydrocarbon receptor and epigenetics

The AhR, a ligand-activated transcription factor, functions beyond the direct regulation of protein-coding genes. Recent research has progressively uncovered profound interactions between AhR and the epigenetic regulatory network, primarily occurring at two levels: the regulation of non-coding RNA networks and interactions with the chromatin modification machinery.

At the level of non-coding RNA regulation, studies indicate that AhR can broadly influence the epigenetic landscape of gene expression. For instance, research has shown that TCDD-activated AhR regulates the expression of a large number of long non-coding RNAs (lncRNAs) in the liver in a dose- and cell type-specific manner (Post et al., 2025). More importantly, studies have revealed a complete AhR-miRNA-mRNA regulatory axis: in a high-fat diet model, activated AhR directly binds to and upregulates the expression of miR-132, which subsequently inhibits its target gene SIRT1 (a crucial deacetylase), ultimately promoting hepatic lipid deposition (Tao et al., 2025). This finding not only establishes AhR as an upstream regulator of epigenetic modulation but also effectively links environmental signals to metabolic epigenetic regulation. At the chromatin level, AhR directly participates in chromatin remodeling by recruiting transcriptional complexes. Research found that resveratrol can inhibit the transcriptional activation of genes like CYP1A1 and CYP1B1 by preventing the recruitment of the AhR/ARNT complex and RNA polymerase II to their regulatory regions (Gao et al., 2016). This mechanistically demonstrates that AhR’s function relies on its binding to specific chromatin sites and the recruitment of the transcriptional machinery, a process that can itself be targeted for epigenetic intervention.

Although these findings preliminarily outline the connection between AhR and epigenetic regulation, this field still faces significant knowledge gaps. A comprehensive “epigenetic map” of AhR’s functional network in the liver is lacking. Key unresolved questions include: How does AhR achieve cell-specific regulation of non-coding RNAs? What are the dynamic changes in AhR’s chromatin binding landscape and associated histone modifications during the transition from acute adaptation to chronic injury? Exploring these directions will be crucial for fully understanding AhR’s complex role in liver pathobiology and for developing novel epigenetic-based therapeutic strategies.

## 3 Conclusion and future perspectives

This review synthesizes the complex role of the AhR in liver injury, revealing its function as a “double-edged sword” in hepatic pathophysiology. Current evidence demonstrates that AhR profoundly influences the progression of liver injury by regulating key processes including apoptosis, stress response, metabolic dysregulation, autophagy, fibrosis, and carcinogenesis. However, its functional output exhibits remarkable context-dependence: it acts as an anti-fibrotic factor in hepatic stellate cells, manifests tumor-suppressive functions during early hepatocarcinogenesis, yet frequently promotes liver injury upon exposure to environmental pollutants. This functional heterogeneity is primarily governed by the interplay of ligand properties, cellular microenvironment, and disease stage.

Although significant progress has been made in understanding AhR’s mechanisms in liver injury, resolving the following key scientific questions will be the focus of future research:

First, elucidating the precise molecular mechanisms underlying AhR’s functional switch represents a core challenge. A deeper understanding of the key molecular switches—including specific co-activators/co-repressors, post-transcriptional modifications, and epigenetic mechanisms—that regulate AhR’s transcriptional activity and target gene selectivity under different pathological states is required. Employing hepatocyte-specific genetic manipulation models and lineage tracing technologies to map the dynamic functional landscape of AhR during the progression of liver diseases will provide crucial insights into its role transition.

Second, revealing the cell-specific networks of AhR signaling demands in-depth investigation. The liver comprises multiple cell types, yet our understanding of AhR’s specific functions in different cells remains limited. Future studies should utilize advanced technologies like single-cell sequencing and spatial transcriptomics, combined with cell-specific knockout models, to precisely delineate the unique action networks of AhR in hepatocytes, cholangiocytes, hepatic stellate cells, and immune cells, as well as their intercellular communication mechanisms.

Third, the structural basis and functional consequences of ligand-specific effects require systematic clarification. Why do different AhR ligands elicit distinct biological outcomes? Integrating structural biology, computational chemistry, and functional genomics to elucidate how ligand-induced conformational changes in AhR differentially recruit downstream effector molecules will provide a theoretical foundation for developing selective AhR modulators.

Finally, advancing the translation of basic research into clinical applications is the ultimate goal. It is essential to validate foundational research findings in clinically relevant models such as human liver tissues, primary cells, and organoids, and to explore the therapeutic potential of AhR-targeting strategies in specific liver disease contexts. Particularly, developing next-generation selective AhR modulators that can precisely modulate specific AhR functions without undesirable side effects holds promise for providing new treatment options for liver fibrosis, metabolic liver diseases, and hepatocellular carcinoma.

In conclusion, the complex role of AhR in liver injury presents both research challenges and rich scientific opportunities. Employing interdisciplinary research strategies to systematically elucidate the regulatory networks governing AhR function will undoubtedly advance our fundamental understanding of liver pathobiology and open new avenues for the precise prevention and treatment of liver diseases.

## References

[B1] Beedanagari S. R. Bebenek I. Bui P. Hankinson O. (2009). Resveratrol inhibits dioxin-induced expression of human CYP1A1 and CYP1B1 by inhibiting recruitment of the aryl hydrocarbon receptor complex and RNA polymerase II to the regulatory regions of the corresponding genes. Toxicol. Sciences an Official Journal Soc. Toxicol. 110 (1), 61–67. 10.1093/toxsci/kfp079 19376845 PMC2696325

[B2] Carambia A. Schuran F. A. (2021). The aryl hydrocarbon receptor in liver inflammation. Seminars Immunopathology 43 (4), 563–575. 10.1007/s00281-021-00867-8 34075438 PMC8443474

[B3] de Tomaso Portaz A. C. Caimi G. R. Sánchez M. Chiappini F. Randi A. S. Kleiman de Pisarev D. L. (2015). Hexachlorobenzene induces cell proliferation, and aryl hydrocarbon receptor expression (AhR) in rat liver preneoplastic foci, and in the human hepatoma cell line HepG2. AhR is a mediator of ERK1/2 signaling, and cell cycle regulation in HCB-treated HepG2 cells. Toxicology 336, 36–47. 10.1016/j.tox.2015.07.013 26219504

[B4] Elbekai R. H. Korashy H. M. Wills K. Gharavi N. El-Kadi A. O. S. (2004). Benzo[a]pyrene, 3-methylcholanthrene and beta-naphthoflavone induce oxidative stress in hepatoma hepa 1c1c7 Cells by an AHR-dependent pathway. Free Radical Research 38 (11), 1191–1200. 10.1080/10715760400017319 15621696

[B5] Fan Y. Boivin G. P. Knudsen E. S. Nebert D. W. Xia Y. Puga A. (2010). The aryl hydrocarbon receptor functions as a tumor suppressor of liver carcinogenesis. Cancer Research 70 (1), 212–220. 10.1158/0008-5472.CAN-09-3090 19996281 PMC2939500

[B6] Gao X. Xie C. Wang Y. Luo Y. Yagai T. Sun D. (2016). The antiandrogen flutamide is a novel aryl hydrocarbon receptor ligand that disrupts bile acid homeostasis in mice through induction of Abcc4. Biochem. Pharmacology 119, 93–104. 10.1016/j.bcp.2016.08.021 27569425 PMC5061623

[B7] Guerrina N. Aloufi N. Shi F. Prasade K. Mehrotra C. Traboulsi H. (2021). The aryl hydrocarbon receptor reduces LC3II expression and controls endoplasmic reticulum stress. Am. Journal Physiology. Lung Cellular Molecular Physiology 320 (3), L339–L355. 10.1152/ajplung.00122.2020 33236922

[B8] Han Q. Yan X. Wang L. Zhang N. Zhang W. Li H. (2023). Aryl hydrocarbon receptor attenuates cholestatic liver injury by regulating bile acid metabolism. Biochem. Biophysical Research Communications 682, 259–265. 10.1016/j.bbrc.2023.10.030 37826949

[B9] Heo M. J. Suh J. H. Lee S. H. Poulsen K. L. An Y. A. Moorthy B. (2023). Aryl hydrocarbon receptor maintains hepatic mitochondrial homeostasis in mice. Mol. Metabolism 72, 101717. 10.1016/j.molmet.2023.101717 37004989 PMC10106517

[B10] Hu Y. Wang R. Diao J. An L. Liu J. Sun D. (2025). Molecular dissection of the AHR-AREG driven EGFR-ERK1/2-CyclinD1 axis in acquired lenvatinib resistance of hepatocellular carcinoma. Biochem. Pharmacology 239, 117032. 10.1016/j.bcp.2025.117032 40482839

[B11] Huang C. Xu X. Wang D. Ma M. Rao K. Wang Z. (2018). The aryl hydrocarbon receptor (AhR) activity and DNA-damaging effects of chlorinated polycyclic aromatic hydrocarbons (Cl-PAHs). Chemosphere 211, 640–647. 10.1016/j.chemosphere.2018.07.087 30098559

[B12] Kim H. R. Kang S. Y. Kim H. O. Park C. W. Chung B. Y. (2020). Role of aryl hydrocarbon receptor activation and autophagy in psoriasis-related inflammation. Int. Journal Molecular Sciences 21 (6), 2195. 10.3390/ijms21062195 32235789 PMC7139675

[B13] Kunitomi C. Harada M. Kusamoto A. Azhary J. M. Nose E. Koike H. (2021). Induction of aryl hydrocarbon receptor in granulosa cells by endoplasmic reticulum stress contributes to pathology of polycystic ovary syndrome. Mol. Human Reproduction 27 (3), gaab003. 10.1093/molehr/gaab003 33493289

[B14] Lahvis G. P. Lindell S. L. Thomas R. S. McCuskey R. S. Murphy C. Glover E. (2000). Portosystemic shunting and persistent fetal vascular structures in aryl hydrocarbon receptor-deficient mice. Proc. Natl. Acad. Sci. U. S. A. 97 (19), 10442–10447. 10.1073/pnas.190256997 10973493 PMC27043

[B15] Lai D. Liu S. Karlsson A. I. Lee W. Wang K. Chen Y. (2014). The novel aryl hydrocarbon receptor inhibitor biseugenol inhibits gastric tumor growth and peritoneal dissemination. Oncotarget 5 (17), 7788–7804. 10.18632/oncotarget.2307 25226618 PMC4202161

[B16] Li B. Liang X. Li Y. Wang R. Wei Y. Liu Q. (2025). Tryptophan catabolites from microbiota ameliorate immune-mediated hepatitis through activating aryl hydrocarbon receptor of T cells. Gut Microbes 17 (1), 2557979. 10.1080/19490976.2025.2557979 40995824 PMC12477874

[B17] Liu S. (2024). Aryl hydrocarbon receptor alleviates hepatic fibrosis by inducing hepatic stellate cell ferroptosis. J. Cellular Molecular Medicine 28 (23), e70278. 10.1111/jcmm.70278 39654034 PMC11628353

[B18] Liu Q. Zhang L. Allman E. L. Hubbard T. D. Murray I. A. Hao F. (2021). The aryl hydrocarbon receptor activates ceramide biosynthesis in mice contributing to hepatic lipogenesis. Toxicology 458, 152831. 10.1016/j.tox.2021.152831 34097992 PMC8327397

[B19] Liu X. Zhang X. Zhu J. Zou W. Liang L. Zhang J. (2025). BbF-induced liver injury in Balb/c mice: AhR activation as the conductor of metabolism, oxidative stress, lipid metabolism disorder, and inflammatory response. Free Radical Biology and Medicine 241, 617–630. 10.1016/j.freeradbiomed.2025.10.008 41061939

[B20] Moreno-Marín N. Merino J. M. Alvarez-Barrientos A. Patel D. P. Takahashi S. González-Sancho J. M. (2018). Aryl hydrocarbon receptor promotes liver polyploidization and inhibits PI3K, ERK, and Wnt/β-Catenin signaling. iScience 4, 44–63. 10.1016/j.isci.2018.05.006 30240752 PMC6147018

[B21] Neuschäfer-Rube F. Schraplau A. Schewe B. Lieske S. Krützfeldt J. Ringel S. (2015). Arylhydrocarbon receptor-dependent mIndy (Slc13a5) induction as possible contributor to benzo[a]pyrene-induced lipid accumulation in hepatocytes. Toxicology 337, 1–9. 10.1016/j.tox.2015.08.007 26303333

[B22] Oh S. J. Im S. Kang S. Lee A. G. Lee B. C. Pak Y. K. (2025). Hepatic AhR activation by TCDD induces obesity and steatosis *via* hepatic plasminogen activator Inhibitor-1 (PAI-1). Int. Journal Molecular Sciences 26 (17), 8452. 10.3390/ijms26178452 40943373 PMC12428968

[B23] Ohashi H. Nishioka K. Nakajima S. Kim S. Suzuki R. Aizaki H. (2018). The aryl hydrocarbon receptor-cytochrome P450 1A1 pathway controls lipid accumulation and enhances the permissiveness for hepatitis C virus assembly. J. Biological Chemistry 293 (51), 19559–19571. 10.1074/jbc.RA118.005033 30381393 PMC6314116

[B24] Ozeki J. Uno S. Ogura M. Choi M. Maeda T. Sakurai K. (2011). Aryl hydrocarbon receptor ligand 2,3,7,8-tetrachlorodibenzo-p-dioxin enhances liver damage in bile duct-ligated mice. Toxicology 280 (1-2), 10–17. 10.1016/j.tox.2010.11.003 21095216 PMC3011047

[B25] Park J. Mangal D. Frey A. J. Harvey R. G. Blair I. A. Penning T. M. (2009). Aryl hydrocarbon receptor facilitates DNA strand breaks and 8-oxo-2'-deoxyguanosine formation by the aldo-keto reductase product benzo[a]pyrene-7,8-dione. J. Biological Chemistry 284 (43), 29725–29734. 10.1074/jbc.M109.042143 19726680 PMC2785604

[B26] Pierre S. Chevallier A. Teixeira-Clerc F. Ambolet-Camoit A. Bui L. Bats A. (2014). Aryl hydrocarbon receptor-dependent induction of liver fibrosis by dioxin. Toxicol. Sciences an Official Journal Soc. Toxicol. 137 (1), 114–124. 10.1093/toxsci/kft236 24154488

[B27] Post G. A. Cholico G. N. Nault R. Zacharewski T. (2025). Cell type- and species-specific regulation of hepatic lncRNAs by TCDD-activated aryl hydrocarbon receptor. Sci. Reports 15 (1), 37322. 10.1038/s41598-025-21357-z 41136526 PMC12552753

[B28] Puga A. Marlowe J. Barnes S. Chang C. Maier A. Tan Z. (2002). Role of the aryl hydrocarbon receptor in cell cycle regulation. Toxicology 181-182, 171–177. 10.1016/s0300-483x(02)00276-7 12505305

[B29] Schuran F. A. Lommetz C. Steudter A. Ghallab A. Wieschendorf B. Schwinge D. (2021). Aryl hydrocarbon receptor activity in hepatocytes sensitizes to hyperacute acetaminophen-induced hepatotoxicity in mice. Cell. Molecular Gastroenterology Hepatology 11 (2), 371–388. 10.1016/j.jcmgh.2020.09.002 32932016 PMC7779786

[B30] Shen D. Dalton T. P. Nebert D. W. Shertzer H. G. (2005). Glutathione redox state regulates mitochondrial reactive oxygen production. J. Biological Chemistry 280 (27), 25305–25312. 10.1074/jbc.M500095200 15883162

[B31] Shin H. S. Lee H. J. Pyo M. C. Ryu D. Lee K. (2019). Ochratoxin A-Induced hepatotoxicity through phase I and phase II reactions regulated by AhR in liver cells. Toxins 11 (7), 377. 10.3390/toxins11070377 31261931 PMC6669489

[B32] Takeda T. Komiya Y. Koga T. Ishida T. Ishii Y. Kikuta Y. (2017). Dioxin-induced increase in leukotriene B4 biosynthesis through the aryl hydrocarbon receptor and its relevance to hepatotoxicity owing to neutrophil infiltration. J. Biol. Chem. 292 (25), 10586–10599. 10.1074/jbc.M116.764332 28487374 PMC5481565

[B33] Tanos R. Murray I. A. Smith P. B. Patterson A. Perdew G. H. (2012). Role of the Ah receptor in homeostatic control of fatty acid synthesis in the liver. Toxicol. Sciences an Official Journal Soc. Toxicol. 129 (2), 372–379. 10.1093/toxsci/kfs204 22696238 PMC3491957

[B34] Tao L. Li X. Zhao M. Shi J. Ji S. Jiang W. (2021). Chrysene, a four-ring polycyclic aromatic hydrocarbon, induces hepatotoxicity in mice by activation of the aryl hydrocarbon receptor (AhR). Chemosphere 276, 130108. 10.1016/j.chemosphere.2021.130108 33711793

[B35] Tao Q. Xie J. Zhou Y. Wu Y. Xia H. Wang J. (2025). High-fat diet mediates the formation of nonalcoholic fatty liver by modulating the AhR/miR-132/SIRT1 axis. J. Nutritional Biochemistry 148, 110174. 10.1016/j.jnutbio.2025.110174 41203141

[B36] Tappenden D. M. Lynn S. G. Crawford R. B. Lee K. Vengellur A. Kaminski N. E. (2011). The aryl hydrocarbon receptor interacts with ATP5α1, a subunit of the ATP synthase complex, and modulates mitochondrial function. Toxicol. Applied Pharmacology 254 (3), 299–310. 10.1016/j.taap.2011.05.004 21616089 PMC3133825

[B37] Tsai C. Li C. Cheng Y. Lee C. Liao P. Lin C. (2017). The inhibition of lung cancer cell migration by AhR-regulated autophagy. Sci. Reports 7, 41927. 10.1038/srep41927 28195146 PMC5307309

[B38] Volz D. C. Kullman S. W. Howarth D. L. Hardman R. C. Hinton D. E. (2008). Protective response of the Ah receptor to ANIT-induced biliary epithelial cell toxicity in see-through medaka. Toxicol. Sciences an Official Journal Soc. Toxicol. 102 (2), 262–277. 10.1093/toxsci/kfm308 18187559

[B39] Walisser J. A. Glover E. Pande K. Liss A. L. Bradfield C. A. (2005). Aryl hydrocarbon receptor-dependent liver development and hepatotoxicity are mediated by different cell types. Proc. Natl. Acad. Sci. U. S. A. 102 (49), 17858–17863. 10.1073/pnas.0504757102 16301529 PMC1308889

[B40] Wang H. Zhou Y. Huang S. (2017). SHP-2 phosphatase controls aryl hydrocarbon receptor-mediated ER stress response in mast cells. Archives Toxicology 91 (4), 1739–1748. 10.1007/s00204-016-1861-1 27709270

[B41] Xu S. Weerachayaphorn J. Cai S. Soroka C. J. Boyer J. L. (2010). Aryl hydrocarbon receptor and NF-E2-related factor 2 are key regulators of human MRP4 expression. Am. Journal Physiology. Gastrointest. Liver Physiology 299 (1), G126–G135. 10.1152/ajpgi.00522.2010 20395535 PMC2904108

[B42] Yan J. Tung H. Li S. Niu Y. Garbacz W. G. Lu P. (2019). Aryl hydrocarbon receptor signaling prevents activation of hepatic stellate cells and liver fibrogenesis in mice. Gastroenterology 157 (3), 793–806.e14. 10.1053/j.gastro.2019.05.066 31170413 PMC6707837

[B43] Yao L. Wang C. Zhang X. Peng L. Liu W. Zhang X. (2016). “Hyperhomocysteinemia activates the aryl hydrocarbon receptor/CD36 pathway to promote hepatic steatosis in mice,”Hepatology, 64 (1), 92–105. 10.1002/hep.28518 26928949

[B44] Zhang Z. Gao Z. Hu W. Yin S. Wang C. Zang Y. (2013). 3,3'-Diindolylmethane ameliorates experimental hepatic fibrosis *via* inhibiting miR-21 expression. Br. Journal Pharmacology 170 (3), 649–660. 10.1111/bph.12323 23902531 PMC3792002

[B45] Zhang W. Xie H. Q. Li Y. Zhou M. Zhou Z. Wang R. (2022). The aryl hydrocarbon receptor: a predominant mediator for the toxicity of emerging dioxin-like compounds. J. Hazardous Materials 426, 128084. 10.1016/j.jhazmat.2021.128084 34952507 PMC9039345

[B46] Zhang M. Lv X. Wang C. Wang L. Wang H. Wang X. (2025). Molecular mechanisms of dose-dependent regulation of hepatic lipid metabolism by BaP through modulation of AhR binding to XRE1 or XRE3. Front. Pharmacology 16, 1595566. 10.3389/fphar.2025.1595566 40786047 PMC12331770

